# **Chronic Arsenic Exposure and Angiogenesis in Human Bronchial Epithelial Cells via the ROS/miR-199a-5p/HIF-1**α**/COX-2 Pathway**

**DOI:** 10.1289/ehp.1307545

**Published:** 2014-01-10

**Authors:** Jun He, Min Wang, Yue Jiang, Qiudan Chen, Shaohua Xu, Qing Xu, Bing-Hua Jiang, Ling-Zhi Liu

**Affiliations:** 1 State Key Lab of Reproductive Medicine, Department of Pathology, Nanjing Medical University, Nanjing, People’s Republic of China; 2 Department of Pathology, Anatomy and Cell Biology, Thomas Jefferson University, Philadelphia, Pennsylvania, USA; 3 Faculty of Software, College of Life Sciences, Fujian Normal University, People’s Republic of China; 4 Changzhou Maternal and Child Care Hospital, Changzhou, People’s Republic of China

## Abstract

Background: Environmental and occupational exposure to arsenic is a major public health concern. Although it has been identified as a human carcinogen, the molecular mechanism underlying the arsenic-induced carcinogenesis is not well understood.

Objectives: We aimed to determine the role and mechanisms of miRNAs in arsenic-induced tumor angiogenesis and tumor growth.

Methods: We utilized an *in vitro* model in which human lung epithelial BEAS-2B cells were transformed through long-term exposure to arsenic. A human xenograft tumor model was established to assess tumor angiogenesis and tumor growth *in vivo*. Tube formation assay and chorioallantoic membranes assay were used to assess tumor angiogenesis.

Results: We found that miR-199a-5p expression levels were more than 100-fold lower in arsenic-transformed cells than parental cells. Re-expression of miR-199a-5p impaired arsenic-induced angiogenesis and tumor growth through its direct targets HIF-1α and COX-2. We further showed that arsenic induced COX-2 expression through HIF-1 regulation at the transcriptional level. In addition, we demonstrated that reactive oxygen species are an upstream event of miR-199a-5p/ HIF-1α/COX-2 pathway in arsenic-induced carcinogenesis.

Conclusion: The findings establish critical roles of miR-199a-5p and its downstream targets HIF-1/COX-2 in arsenic-induced tumor growth and angiogenesis.

Citation: He J, Wang M, Jiang Y, Chen Q, Xu S, Xu Q, Jiang BH, Liu LZ. 2014. Chronic arsenic exposure and angiogenesis in human bronchial epithelial cells via the ROS/miR-199a-5p/HIF-1α/COX-2 Pathway. Environ Health Perspect 122:255–261; http://dx.doi.org/10.1289/ehp.1307545

## Introduction

Arsenic is a widely distributed semimetallic element that occurs naturally in various compounds in the crust of the earth. Approximately 160 million people worldwide are exposed to unsafe high levels of arsenic in drinking water (Hubaux et al. 2013). In particular, about 25 million people in Bangladesh and 6 million in India are chronically exposed to a very high level of arsenic exceeding 50 μg/L via groundwater (Rahman et al. 2001), which is significantly greater than the current maximum contaminant level (MCL) of 10 μg/L set forth by the U.S. Environmental Protection Agency (2014). Long-term arsenic exposure leads to an increased risk of skin, bladder, and liver cancers and, especially, lung cancer. Epidemiological investigations and laboratory studies have provided evidence that ingestion of arsenic via drinking water or inhalation of air increases the risk of lung cancer in a dose–dependent way (Boffetta 2004; Chen et al. 2004). However, the exact mechanism of arsenic-induced cell malignant transformation and cancer development remains to be investigated.

Current proposed mechanisms underlying arsenic carcinogenesis include arsenic-induced genetic changes and epigenetic alterations (Hubaux et al. 2013). The epigenetic alterations consist of histone modification, DNA methylation, and microRNA (miRNA) regulation. We and others (Beezhold et al. 2011; He et al. 2013b; Wang et al. 2011) have observed aberrant miRNA expression profiles after heavy-metal exposure in various cell types, which indicates the involvement of miRNAs in environmental carcinogenesis. However, it remains to be determined whether these miRNAs play causal roles in arsenic-induced tumor initiation and development.

Angiogenesis is the formation of new blood vessels from existing vasculatures to induce tumor growth. Outside of its role in physiological processes such as embryo development and wound healing, angiogenesis is required for cancer development. Angiogenesis is critical for a tumor colony to grow and become invasive (D’Amico 2004). In the present study, we aimed to determine *a*) the roles of miRNAs in arsenic-induced tumor angiogenesis and tumor growth, *b*) the direct targets of miRNAs in regulating tumorigenesis, and *c*) the functional relevant angiogenesis factors in arsenic- and miRNA-mediated carcinogenesis.

## Materials and Methods

*Cell culture and generation of stable cell lines*. Human bronchial epithelial BEAS-2B cells [American Type Culture Collection (ATCC), Manassas, VA, USA] were cultured in DMEM (Dulbecco’s modiﬁed Eagle’s medium; Invitrogen, Carlsbad, CA, USA) supplemented with 10% fetal bovine serum (FBS). Human umbilical vein endothelial cells (HUVEC) (purchased from ATCC) were cultured in EBM-2 (endothelial basal medium-2) complete medium. Arsenic-transformed BEAS-2B (AsT) cells (Carpenter et al. 2011) stably overexpressing miR-199a or miR-control were generated by infecting BEAS-2B cells with lentivirus carrying miR-199a-RFP or a negative control precursor (Applied Biosystems, Carlsbad, CA, USA), followed by selection with puromycin. To establish stable cell lines overexpressing COX-2 (cyclooxygenase-2), 293T cells were transfected with lentivirus carrying the *COX-2* plasmid (GeneCopoeia, Rockville, MD, USA) or an empty vector to generate infectious virus. Then AsT cells were transduced with virus, followed by the puromycin selection. We also prepared PI3K (phosphatidylinositide 3-kinase)–transformed chicken embryo fibroblast cells as was previously described (Chang et al. 1997; Jiang et al. 2000).

*Animal experiment*. Female CrTac:NCr-*Foxn1^nu^* mice (8 weeks of age) were purchased from Taconic (Hudson, NY, USA), and maintained in pathogen-free conditions. Animals were housed in sterilized cages (5 mice/cage) with hardwood chip bedding. Standardized commercial diets were provided, and sterilized water was available at all times. The average weight of animals on arrival was 20 ± 2 g (mean ± SD). A total of 2 × 10^6^ AsT/miR-cont cells or AsT/miR-199a cells (AsT cells stably overexpressing miR-control or miR-199a, respectively) in 80 μL were injected subcutaneously into the flanks of nude (*nu/nu*) mice (*n* = 10/group). The animals used in research were treated humanely according to the Institutional Animal Care and Use Committee of Thomas Jefferson University. The mice were euthanized by decapitation 6 weeks after injection. Tumor tissues were removed and weighed. Parts of tissues were paraffin-embedded, and other parts were snap-frozen in liquid nitrogen and stored at –80°C for immunohistochemical analysis.

*Reagents and antibodies*. Sodium arsenic, catalase, and H_2_O_2_ were purchased from Sigma-Aldrich (St. Louis, MO, USA). Small interfering RNA (siRNA) Smartpools (pools of four individual siRNAs) against COX-2, HIF-1α (hypoxia inducible factor 1, alpha), and a scrambled control were purchased from Dharmacon (Lafayette, CO, USA). COX-2 antibody was from Cell Signaling Technology (Beverly, MA, USA). CD31 (platelet/endothelial cell adhesion molecule 1) antibody for analyzing paraffin-embedded tissues was from Santa Cruz Biotechnology (Santa Cruz, CA, USA) and for analyzing frozen tissues, from BD Pharmingen (San Jose, CA, USA). HIF-1α antibody was from BD Biosciences (Franklin Lakes, NJ, USA), and α-SMA (smooth muscle α-actin) antibody was obtained from Abcam (Cambridge, MA, USA). Primary antibodies used for Western analysis were diluted 1:1,000 in 5% bovine serum albumin as a working concentration and were incubated on a shaker overnight at 4°C.

*RT-qPCR analysis*. Total RNAs were extracted using Trizol (Life Technologies, Carlsbad, CA, USA). The synthesis of cDNA was performed using oligo (dT)18 primers and M-MLV (Moloney Murine Leukemia Virus) reverse transcriptase (Promega, Madison, WI, USA). The amplification was performed by polymerase chain reaction (PCR). SYBR-green reverse transcription PCR (RT-qPCR) was performed to detect *COX-2* and *GADPH* (glyceraldehyde-3-phosphate dehydrogenase) mRNA levels using the Power SYBR Green PCR Master Mix Kit (Applied Biosystems). Taqman RT-qPCR was performed to detect miRNA expression levels using the Taqman miRNA Reverse Transcription kit and Taqman universal PCR Master Mix (Applied Biosystems). The sequences of primer used for SYBR-green RT-qPCR were as follows:

*COX*-2 forward: 5´-TCAGC​CAT​ACAGC​AAA​TCCT​T-3´
*COX*-2 reverse: 5´-CTGC​ACTG​TGTT​AGTG​G-3´
*GAPDH* forward: 5´-ATGG​GTGT​GAAC​CATGA GAAG​TATG​-3´
*GADPH* reverse: 5´GGTG​CAGG​AGGC​ATTG​CT-3´.

*Immunohistochemistry and immunofluorescence*. Paraffin-embedded tissue sections and frozen tissue sections were prepared by routine methods (Fero Lab, Fred Hutchinson Cancer Research Center 2011). For immunohistochemistry, the Dako Envision two-step method of immunohistochemistry was used to stain CD31 (1:100 dilution) and α-SMA (1:150 dilution) in xenograft tumor tissues as described previously (He et al. 2012a). Tissue sections were incubated with primary antibodies in a humid chamber overnight at 4°C. The microvessel density reflected by CD31-positive staining was counted in three different fields per section. For immunofluorescence staining, frozen sections were incubated with primary antibodies overnight. Goat anti-rabbit or anti-mouse IgG conjugated with fluorescein isothiocyanate or Texas Red (both from Santa Cruz Biotechnology) (1:200 dilution) were used as secondary antibodies and incubated for 2 hr at room temperature. Slides were mounted with anti-fade DAPI reagent (Invitrogen, Grand Island, NY, USA).

*Chromatin immunoprecipation (ChIP)–quantitative PCR (qPCR).* ChIP-qPCR was performed using the EpiTect ChIP OneDay Kit (QIAGEN, Valencia, CA, USA) according to the manufacturer’s instructions. HIF-1α antibody (Abcam) was used to pull down the protein–chromatin complexes. Rabbit IgG was used as a negative control. The immunoprecipated DNA was quantified using SYBR Green qPCR (Applied Biosystems). All results were normalized to 1% input value of the same sample. COX-2 primers flanking the hypoxia-response elements (HRE) for SYBR Green qPCR were as follows:

Forward: 5´-TATA​CAGC​CTAT​TAAG​CGTC​GTCA​-3´
Reverse: 5´-CGTG​TCTG​GTCT​GTAC​GTCT​TAG-3´.

*Prostaglandin E_2_ (PGE_2_) ELISA*. Cells were plated at 0.1 × 10^6^ cells/well of a 24-well plate and allowed to recover overnight. The following day, the media were replaced with fresh media, and then the cells were cultured in normoxia or hypoxia (1% O_2_) for 24 hr. The conditioned media were then collected and cleared of cellular debris by centrifugation at 2,000 rpm for 2 min. PGE_2_ concentrations were determined using the Prostaglandin E_2_ EIA Kit–Monoclonal ELISA kit (no. 514010; Cayman Chemical, Ann Arbor, MI, USA) according to the manufacturer’s instructions.

*miRNA luciferase reporter constructs and luciferase activity assay*. The 3´UTR-luciferase reporter constructs containing the 3´UTR regions of *COX-2* with wild-type and mutant binding sites of miR-199a were amplified using the PCR method (GoTaq® G2 Flexi DNA Polymerase; Promega) according to the manufacturer’s instructions. The PCR products were cloned into the pMiR-luc luciferase reporter vector (Ambion, Grand Island, NY, USA). The mutant 3´UTR constructs were made by introducing four point mutations into the putative seed regions of COX-2. All the constructs containing 3´UTR inserts were sequenced and verified. The luciferase activity assay was performed as previously described (He et al. 2013a).

*Site-directed mutagenesis*. The human full-length COX-2 reporter used was a generous gift from J. Li (Harvard University, Boston, MA, USA) (Wu et al. 2006). To generate the HRE-mutant *COX-2* reporter, we performed site-directed mutagenesis on the wild-type *COX-2* reporter at the potential HIF-1α binding sites with 3 base pair substitutions as previously described (Jiang et al. 1996). The mutant *COX-2* reporter construct was validated by DNA sequencing.

*Tube formation assay*. HUVEC were cultured in EBM-2 complete medium, and switched to EBM-2 basal medium containing 0.2% FBS for 24 hr before performing the tube formation assay. The conditioned media were prepared from different cells by replacing normal culture medium with serum-reduced medium (1% FBS). After culture for 24 hr, the serum-reduced media were collected and stored at –20°C for later use. The HUVEC were trypsinized, counted, and resuspended in EBM-2 basic medium; then they were mixed with an equal volume of the conditioned medium and seeded on Matrigel-pretreated 96-well plates at 2 × 10^4^ cells/well. After culture for 6-12 hr, tube formation was observed under a light microscope and photographed. The total lengths of the tubes in each well were measured using CellSens Standard software (Olympus; Hamburg, Germany).

*The chorioallantoic membranes (CAM) assay*. White Leghorn fertilized chicken eggs (Charles River, Malvern, PA, USA) were incubated at 37°C under constant humidity. Cells were transfected with miRNA precursors, or treated as specifically indicated in the figure legends. After transfection for 12 hr, the cells were trypsinized, counted, and resuspended in the serum-free medium. The cell suspensions were mixed with Matrigel at a 1:1 ratio, and implanted onto the CAM of chicken eggs on day-9 embryos. Tumor angiogenesis responses were analyzed 5 days after the implantation. The tumor/Matrigel plugs were trimmed off the CAM and photographed. The number of blood vessels as the index of angiogenesis was analyzed by counting the branches of blood vessels in three representative areas (each 1.0 mm^2^) by two observers in a double blind manner.

*miRNA transfection*. Cells were cultured in 6-well plates to reach a 60% confluency, and transfected using miR-199a or a negative-control precursor (both from Applied Biosystems) at 30 nM using Lipofectamine RNAiMAX reagent (Invitrogen, Grand Island, NY, USA) according to the manufacturer’s instructions. Total proteins and RNAs were prepared from the cells 60–70 hr after the transfection, followed by Western blotting or RT-PCR analysis.

*Hypoxia treatment*. A hypoxia incubator chamber (Stemcell, Vancouver, British Columbia, Canada) was used to generate the hypoxia environment for cell culture. Cells were cultured in hypoxia conditions (1% O_2_) for 24 hr at 37°C.

*Reactive oxygen species (ROS) study*. Cells were treated with ROS scavenger catalase (1,500 U) or with H_2_O_2_ (50 μM) for 12 hr. Then cells were harvested, and total proteins were extracted for Western blotting analysis.

*Statistical analysis*. All the results were obtained from at least three independent experiments. Results are presented as mean ± SE and were analyzed by Student’s *t*-test or one-way analyis of variance (ANOVA). All results were analyzed by SPSS for Windows, version 11.5 (IBM, Chicago, IL, USA). Differences were considered significant with *p* < 0.05.

## Results

**Figure 1:**
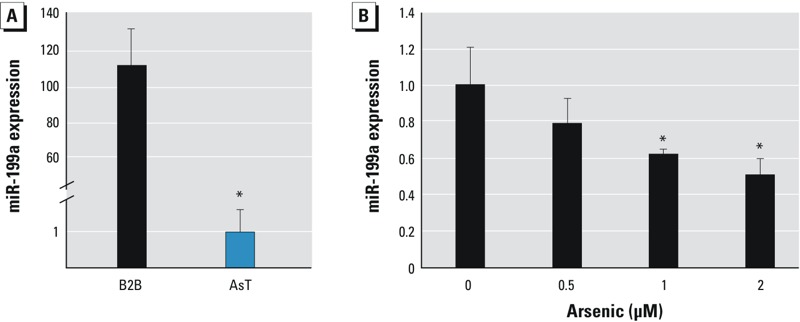
miR-199a is down-regulated in AsT cells. (*A*) miR-199a expression levels were determined by Taqman RT-qPCR in AsT cells and BEAS-2B (B2B) cells. (*B*) B2B cells were treated with sodium arsenic at different concentrations for 24 hr. miR-199a expression levels were determined by RT-qPCR. Data are presented as mean ± SE and analyzed by Student’s *t*-test. *p < 0.05, compared with control cells.

*miR-199a-5p is down-regulated in AsT cells*. In order to investigate the mechanism of arsenic-induced carcinogenesis, we previously established an *in vitro* model by transforming immortalized human lung epithelial BEAS-2B cells via chronic exposure to 1 μM sodium arsenic for 26 weeks (Carpenter et al. 2011). BEAS-2B cells cultured in arsenic-free medium served as a passage-matched control. We performed miRNA microarray analysis to compare the miRNA profiles between parental cells (BEAS-2B) and AsT cells. We found that miR-199a (referred to miR-199a-5p) was the most down-regulated miRNA among the list of miRNAs examined (data not shown). We further validated the result by performing Taqman RT-qPCR analysis. As shown in Figure 1A, miR-199a was 100-fold lower in AsT cells, indicating a major change of miRNA abundance in cell malignant transformation (He et al. 2013b). To investigate the relationship between arsenic treatment and miR-199a expression, we treated BEAS-2B cells with sodium arsenic at the doses of 0.5 μM, 1 μM, and 2 μM for 24 hr. miR-199a expression levels were significantly decreased by arsenic treatment at the dose of ≥ 1 μM (Figure 1B). To determine whether cell transformation affects miR-199a expression, we tested two different types of cell lines transformed by oncogenes: the AsT cells and PI3K–transformed chicken embryo fibroblast cells described above. miR-199a levels were decreased in transformed cells compared with the parental control cells, but the difference was not statistically significant (see Supplemental Material, Figure S1). We also used newly transformed immortalized RE3K rat kidney cells [by N-Ras (neuroblastoma Ras)] (Kolligs et al. 1999) (see Supplemental Material, Figure S2A). We found that miR-199a expression levels in N-Ras–transformed cells were 1.9-fold lower compared with the control cells (see Supplemental Material, Figure S2B), indicating that transformation and/or oncogenes may decrease miR-199a expression. However, given that miR-199a expression levels were more than 100-fold lower in AsT cells, we reason that arsenic plays a major role in the suppression of miR-199a expression.

**Figure 2:**
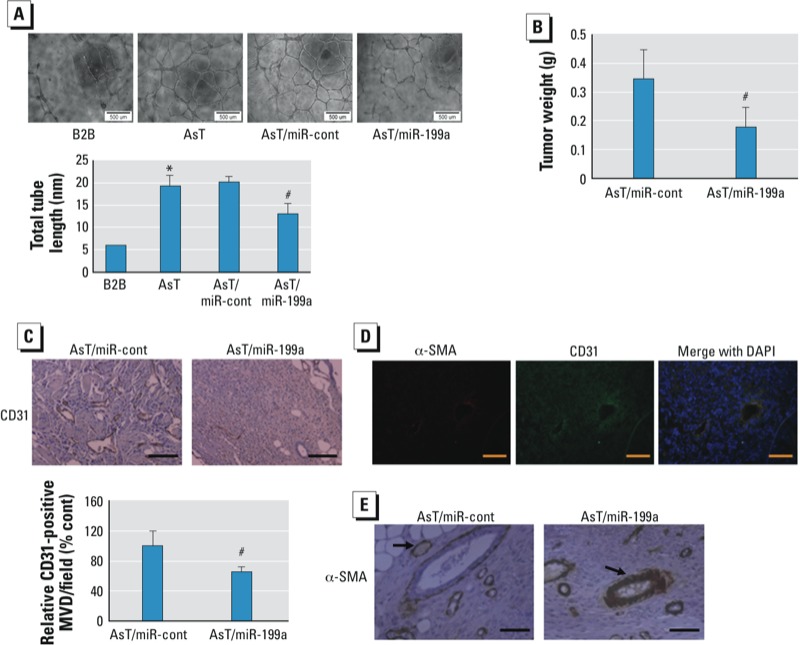
miR-199a inhibits arsenic-induced angiogenesis. (*A*) HUVEC cells were cultured in serum-free medium overnight and resuspended in basic EBM-2 medium. Data represent mean ± SE from six replicates from each treatment and were analyzed by one-way ANOVA. Bar = 500 μM. (*B*) Weight of xenograft tumors from nude mice was measured 6 weeks after cell injection. Data represent mean ± SE (*n* = 10/group). (*C*) Paraffin-embedded tumor tissue sections from both groups were used for immunohistochemical staining using antibodies against CD31. Top: representative sections (magnification: 160×; bar = 50 μM). Bottom: quantification of microvessel density (MVD) indicated by CD31 staining in tumor sections. Data represent mean ± SE from five different tumor sections from each group. (*D*) Frozen tissue sections were used for immunofluorescence staining (magnification: 200×; bar = 50 μM). (*E*) Paraffin-embedded tumor tissue sections were used for immunohistochemical staining using antibodies against α-SMA. Representative sections are shown. Arrow indicates mural cells (magnification: 320×; bar = 50 μM). *p < 0.05, compared with B2B. ^#^*p* < 0.05, compared with AsT/miR-cont.

*miR-199a inhibits arsenic-induced angiogenesis* in vitro *and* in vivo. Our earlier study (He et al. 2013a) showed that miR-199a processes an anti-angiogenic property in ovarian cancer cells. We then investigated the functional effect of miR-199a in arsenic-induced angiogenesis by performing a tube formation assay *in vitro*. As shown in Figure 2A, tube formation was strongly induced when cultured in conditioned medium prepared from AsT cells as compared with its parental control BEAS-2B cells. Transient transfection of miR-199a in AsT cells decreased tube formation by 40%. We next investigated the angiogenic effect of miR-199a *in vivo*. First, we established AsT cells stably expressing miR-199a by transducing lentivirus-carrying miR-199a-RFP. Then we generated xenograft tumors by injecting the stable cells AsT/miR-cont and AsT/miR-199a subcutaneously in nude mice and allowed tumors to grow for 6 weeks. Overexpression of miR-199a decreased the tumor weight by 50% as compared with negative control cells (Figure 2B). The number of microvessels indicated by the endothelial marker CD31 staining in miR-199a–overexpressing tumors was significantly lower than in the control (Figure 2C). As in normal blood vessels, tumor vessels consist of endothelial cells, mural cells (pericytes and smooth muscle cells), and basement membrane (Baluk et al. 2003). The expression of α-SMA was restricted to vascular smooth muscle cells and co-localized with CD31 (Figure 2D). Interestingly, we noticed that blood vessels from AsT/miR-199a tumors had many more mural cells reflected by α-SMA immunoreactivities than AsT/miR-cont tumors, suggesting that miR-199a decreases arsenic-induced tumor angiogenesis but promotes blood vessel maturation (Figure 2E).

**Figure 3:**
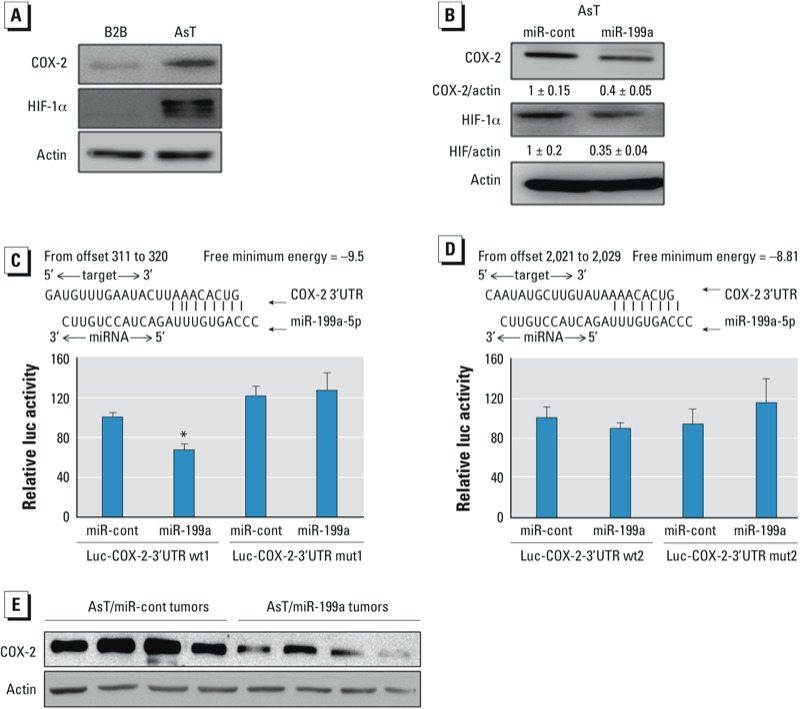
miR-199a directly targets both HIF-1α and COX-2. (*A*) Total proteins prepared from AsT cells and BEAS-2B (B2B) cells were used to determine protein levels of COX and HIF-1α by Western blotting analysis. (*B*) AsT cells were transiently transfected with 25-nM miR-cont or miR-199a precursors for 72 hr, followed by Western blotting analysis. (*C*, *D*) Sequence alignment of human miR-199a sequence with 3´ UTR region of COX-2. Data are presented as mean ± SE and analyzed by Student’s t-test. (*E*) COX-2 protein levels in tumor tissues (*n* = 10/group) were determined by Western blotting analysis. Representative samples are shown. *p < 0.05, compared with control cells.

*miR-199a directly targets both HIF-1*α *and COX-2*. HIF-1 is one of the major pro-angiogenic factors through inducing transcriptional activation of vascular endothelial growth factor (VEGF) (Semenza 2000). Another potent angiogenic activator for tumor angiogenesis is COX-2, which is a rate-limiting enzyme in the conversion of arachadonic acid precursors in the cell membrane into PGE_2_ (Xue and Shah 2013). COX-2 and HIF-1α expression are frequently regulated by similar stimuli and control similar processes within the cells (Gately 2000; Liu et al. 1999). In the present study, we observed that basal levels of HIF-1α and COX-2 under normoxia were markedly up-regulated in AsT cells (Figure 3A). Rane et al. (2009) reported that miR-199a directly targets HIF-1α in cardiac myocytes. Indeed, overexpression of miR-199a in AsT suppressed HIF-1α expression (Figure 3B). Interestingly, we noticed that miR-199a was also capable of down-regulating COX-2 expression. To explore whether COX-2 can be directly targeted by miR-199a, we used the “target search” process to predict possible binding sites and free minimal energy of bindings and found that COX-2 is a putative target of miR-199a with two potential binding regions. *COX-2* 3´UTR luciferase reporters containing two putative miR-199a binding sites were constructed to validate the direct binding between *COX-2* mRNA 3´UTR and miR-199a. Co-transfection of miR-199a precursor with wild-type reporter constructs containing binding sites (*COX-2* 3´UTR 311/320) greatly decreased the luciferase activities in BEAS-2B cells, whereas co-transfection with the corresponding reporter containing point mutant at putative miR-199a binding sites did not affect the luciferase activities (Figure 3C). The luciferase activity in reporters containing putative binding sites in *COX-2* 3´UTR from 2021 to 2029 was not affected by miR-199a, which suggests that this computation-predicted site is not functionally targeted by miR-199a (Figure 3D). Consistently, the expression levels of COX-2 in xenograft tumors were generally lower in AsT/miR-199a groups than those in AsT/miR-cont groups (Figure 3E). These findings demonstrate that COX-2 is a novel direct target of miR-199a.

**Figure 4:**
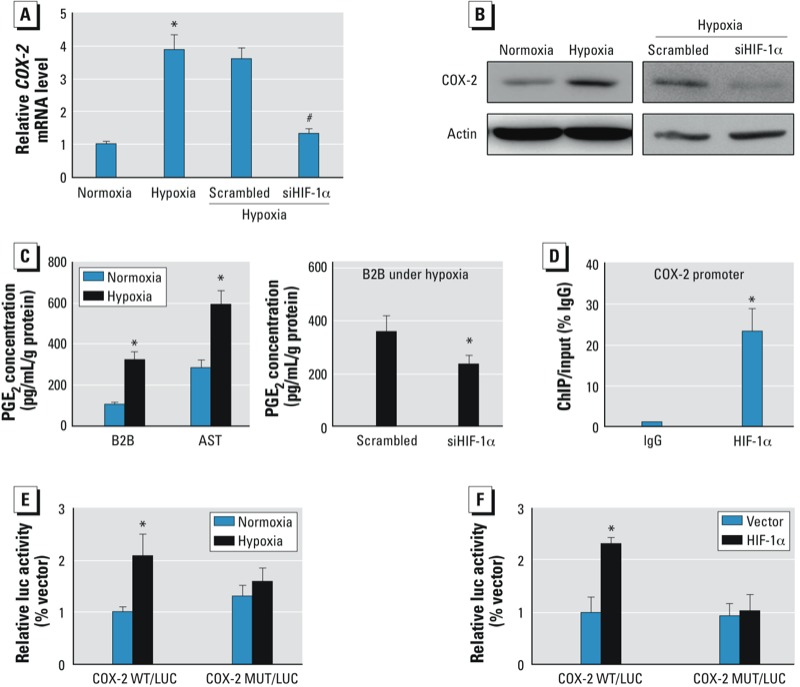
HIF-1α directly regulates COX-2 expression at the transcriptional level. (*A*,*B*) BEAS-2B (B2B) cells were incubated under normoxic or hypoxic condition (1% oxygen) for 24 hr or transfected with 50 nM of a siRNA scramble control or a siRNA SMARTpool against HIF-1α for 72 hr as indicated. The mRNA levels (*A*) and protein levels (*B*) of COX-2 were determined by SYBR-green RT-qPCR or Western blotting analysis, respectively. (*C*) The concentrations of PGE2 in B2B cells and AsT cells were determined by ELISA. (*D*) B2B cells were cultured under hypoxia for 24 hr and were subjected to the ChIP assay using antibodies against IgG or HIF-1α. The antibody-promoter binding signals were analyzed by SYBR-qPCR. (*E*) B2B cells were co-transfected with COX-2 luciferase reporters with wild-type or mutant HIF-1α binding sites and β-gal plasmid. Cells were incubated under normoxia or hypoxia 24 hr before harvest. (*F*) B2B cells were co-transfected with *COX-2* wild-type or mutant reporters, HIF-1α plasmid or vector, and β-gal plasmid. Luciferase activity assay was performed 60 hr after transfection. Data are presented as mean ± SE of three independent experiments and analyzed by one-way ANOVA. *p < 0.05, compared with control. ^#^*p* < 0.05, compared with scrambled control.

*HIF-1*α *directly regulates COX-2 at the transcriptional level*. We previously found that HIF-1α can be strongly induced by acute arsenic treatment in prostate cancer cells under normoxia condition (Gao et al. 2004). In this study, we observed that both HIF-1α and COX-2 expression levels were dramatically up-regulated in AsT cells. We speculate whether there is an interaction between HIF-1α and COX-2 in this context. The COX-2 promoter contains several HREs, one of which we predicted to be functional (Jiang et al. 2009). In the present study, we first investigated whether HIF-1α is required for arsenic-induced COX-2 expression. *COX-2* mRNA and protein levels in BEAS-2B cells were up-regulated under the hypoxia condition, whereas knockdown of HIF-1α by specific siRNA almost completely reversed the induction (Figures 4A-B). This indicates that hypoxia stimulates COX-2 expression via HIF-1α. We further measured the concentration of COX-2 product, PGE_2_, with the same treatments. Both BEAS-2B cells and AsT cells produced more PGE_2_ under hypoxia. Conversely, knockdown of HIF-1α decreased PGE_2_ concentration with hypoxic treatment (Figure 4C). To determine if there is a direct interaction between HIF-1α and COX-2 promoter, we performed the ChIP assay. BEAS-2B cells were cultured in hypoxic chamber for 24h and were subjected to ChIP assay. As shown in Figure 4D, antibody against HIF-1α was able to pull down COX-2 promoter suggesting the association between HIF-1α and *COX-2* gene. The earlier findings in our group predicted that one of HRE (-576/-584) in the *COX-2* promoter would be functional (Jiang et al. 2009). We validated the binding sites by constructing COX-2 promoter luciferase reporters with wild-type or mutant HRE. As shown in Figure 4E, hypoxia stimulated COX-2 transcriptional activity measured by luciferase activity in BEAS-2B cells transfected with wild-type reporter. Conversely, BEAS-2B cells transfected with mutant reporter weren’t affected by hypoxia. Furthermore, co-transfection of HIF-1α with the wild-type reporter similarly promoted COX-2 transcription while co-transfection with the mutant reporter did not affect the luciferase activities (Figure 4F). Collectively, these data support that HIF-1α directly regulates COX-2 at the transcriptional level.

**Figure 5:**
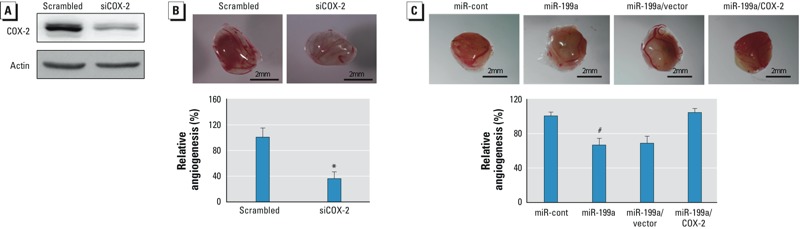
miR-199a inhibits As-induced angiogenesis via COX-2. (*A*) AsT cells were transfected with 50 nM of a siRNA scramble control or smartpool siRNAs against COX-2 for 60 hr, followed by Western blotting analysis. (*B*) The cells were implanted onto the CAMs 24 hr after transfection (AsT cells were transfected with a siRNA control or a siRNAs against COX-2) to perform angiogenesis assay. Top: representative images from each group; bottom: relative angiogenesis responses (mean ± SE; n = 8/group). (*C*) AsT, AsT/vector, or AsT/COX-2 cells were infected with miR-199a lentivirus for 24 hr. Then cells were used to perform angiogenesis assay. Top: representative images. Bottom: relative angiogenesis responses (mean ± SE; n = 8). One-way ANOVA was used to analyze the differences among various groups. *p < 0.01, compared with scrambled control. ^#^*p* < 0.01, compared with miR-cont (AsT cells infected by lentivirus containing miR-cont).

*miR-199a inhibits arsenic-induced angiogenesis via COX-2 expression*. To determine the role of COX-2 in arsenic-induced angiogenesis, we knocked down *COX-2* to evaluate the angiogenesis response using the CAM assay (Figure 5A). We observed a dramatic decrease of angiogenic potential in *COX-2* knockdown cells (Figure 5B). To test whether the overexpression of COX-2 without miR-199a binding sites is able to reverse miR-199a–inhibited angiogensis in AsT cells, we established stable AsT cells overexpressing COX2 by transducing lentivirus carrying COX-2 cDNA without 3´UTR region. We observed that forced expression of COX-2 completely reversed the inhibitory effect of miR-199a in angiogenesis (Figure 5C). Taken together, these results suggest that miR-199a inhibits arsenic-induced angiogenesis by directly targeting COX-2 expression.

**Figure 6:**
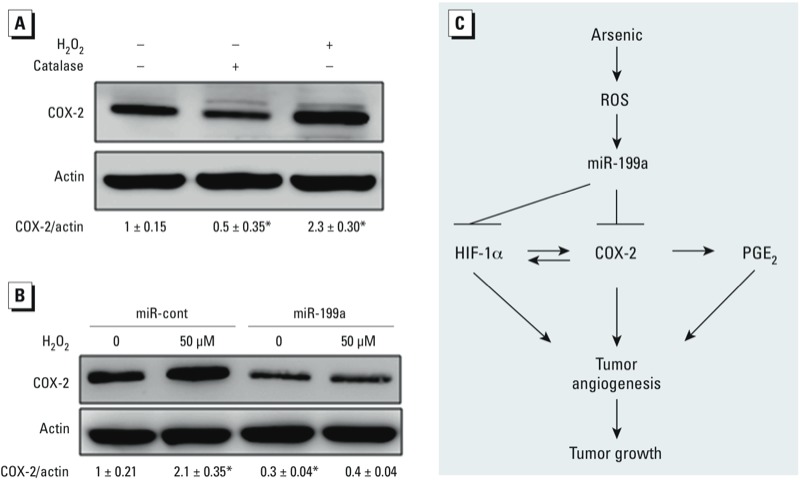
Excessive ROS is an upstream signal of miR-199a/COX-2 pathway in AsT cells. (*A*) AsT cells were treated with catalase (1,500 U) or H_2_O_2_ (50 μM) for 12 hr, followed by Western blotting analysis. Bottom: quantification from three independent experiments using densitometry. (*B*) AsT cells stably expressing control or miR-199a were treated with H_2_O_2_ (50 μM) for 12 hr. COX-2 expression levels were determined by Western blotting analysis. Bottom: quantification from three independent experiments using densitometry. Student’s t-test was used to determine the difference. (*C*) Schematic diagram for proposed pathway. *p < 0.05, compared with control.

*ROS induce COX-2 pathway by suppressing miR-199a in AsT cells*. Induction of ROS in cells is closely related with heavy metal exposure including arsenic, chromium, and cadmium (Azad et al. 2010; Huang et al. 2004; Wang et al. 2012). We previously observed that AsT cells produced higher ROS (Carpenter et al. 2011). In addition, our earlier findings indicate that miR-199a is a ROS responsive miRNA in which ROS inhibit miR-199a expression through increasing the promoter methylation of miR-199a gene by DNA methyltransferase 1 (He et al. 2012b). In this study, we found that ROS treatment increased COX-2 expression, whereas the ROS scavenger catalase decreased COX-2 expression (Figure 6A). To investigate whether ROS are upstream signals for inhibiting miR-199a expression, we treated AsT cells stably overexpressing miR-199a and AsT control cells with H_2_O_2_. We found that H_2_O_2_–induced COX-2 expression in AsT/miR-cont cells, but no induction was observed in AsT/miR-199a cells (Figure 6B).

## Discussion

The strong links between the aberration of miRNAs profiles and carcinogenesis, cancer development, prognosis and chemoresistance have been confirmed by a plethora of bench and clinical studies (reviewed by Iorio and Croce 2012). In this study, we identified that exposure to arsenic induced the suppression of miR-199a expression, which led to increased angiogenesis responses and tumor growth.

The process of angiogenesis normally occurs in the embryo, placenta, and during menstrual cycle and wound-healing (Patella and Rainaldi 2012). However, under pathological situations such as cancer, the same angiogenic signaling pathways in tumors are induced in order to obtain the sufficient blood supply for tumor growth. A number of miRNAs have been reported to be involved in blood vessel development and angiogenesis by directly or indirectly regulating pro-angiogenic factors or anti-angiogenic factors (Hua et al. 2006; Poliseno et al. 2006; Urbich et al. 2008). Our previous study (He et al. 2013a) showed that miR-199a inhibits tumor angiogenesis by targeting ERBB2 and ERBB3 thus suppressing their downstream VEGF in ovarian cancer cells. In the context of arsenic-induced transformation, miR-199a showed strong anti-angiogenic properties not only by directly targeting HIF-1α but also another proangiogenic factor COX-2, which highlights the important role of miR-199a in angiogenesis. Tumor vessels are leaky, immature, or morphologically abnormal because of the absent or incomplete basement membrane and mural cells (pericytes and smooth muscle cells). Failure of tumor vessels to recruit a normal coat of mural cells may contribute to the abnormality of vessels (Abramsson et al. 2002). Berthod et al. (2012) showed that α-SMA expressing cells were present around capillaries and can thus act as a pericyte marker. Interestingly, we found that miR-199a can not only reduce the total number of microvessels in tumors but also promote vascular maturation as indicated by the higher α-SMA staining.

HIF-1α signaling plays a critical role in tumor metastasis, invasion, metabolism, and, especially, angiogenesis (Covello and Simon 2004; Lum et al. 2007). We found that the baseline of HIF-1α expression, which is oxygen independent, was dramatically up-regulated in arsenic-transformed cells. The underlying mechanism might be the activation of thymoma viral oncogene (*AKT*), mitogen-activated protein kinase (*ERK*), mechanistic target of rapamycin (serine/threonine kinase; *mTOR*), and ribosomal protein S6 kinase, polypeptide 1 (*p70S6K1*) genes, as suggested by our earlier results (Carpenter et al. 2011). A recent study reported that overexpression of miR-199a inhibited the hypoxia-induced cell proliferation in non-small cell lung cancer by suppressing HIF-1α (Ding et al. 2013). Oltipraz, a cancer chemopreventive agent, has anti-angiogenic property mediated by miR-199a induction and HIF-1α inhibition (Kang et al. 2012). Consistent with the report of Kang et al. (2012), our results show that miR-199a suppression by arsenic exposure releases its direct target inhibition of HIF-1α. More importantly, we also validated that COX-2 is a novel target of miR-199a and is functional in AsT cells. The COX-2 expression was almost undetectable in parental BEAS-2B cells, but it was pronouncedly expressed after chronic arsenic exposure. Our functional study revealed that COX-2 plays an important role in arsenic-induced angiogenesis, which can be impaired by miR-199a overexpression. Furthermore, consistent with other studies (Csiki et al. 2006; Kaidi et al. 2006), both protein-DNA binding assay and luciferase reporter assay confirmed that HIF-1α directly regulates COX-2 expression in parental BEAS-2B cells. This result along with data that PGE_2_ can stimulate VEGF expression through the activation of HIF-1α (Greenhough et al. 2009) suggests a positive-feedback between HIF-1α and COX-2 in arsenic-induced angiogenesis. The findings also indicate that miR-199a may have high therapeutic efficiency as a tumor suppressor by targeting both HIF-1α and COX-2. In addition, miR-199a-3p has also been reported to target COX-2 in human chondrocytes (Akhtar and Haqqi 2012). Because miR-199a-3p and miR-199a-5p (miR-199a) are derived from the same precursor, the expression levels of both miRNAs may be somewhat regulated by the same mechanism(s). Further investigation of the upstream molecules that may indirectly modulate COX-2 expression via both miRNAs in the future is of interest.

Overproduction of ROS is closely related to heavy metal–mediated carcinogenesis (Lee et al. 2012). Oxidative stress was reported as a mediator of arsenic-induced cell transformation and carcinogenesis (Carpenter et al. 2011; Wang et al. 2012). In addition, ROS can activate HIF-1α and COX-2 in various contexts (Bonello et al. 2007; Chen et al. 2012). However, the mechanism underlying how ROS regulate HIF-1α and COX-2 remains to be investigated. Here, we provide a link in which repression of miR-199a by arsenic-induced ROS activates HIF-1α and COX-2 expression.

We found similar miR-199a suppression and HIF-1α/COX-2 activation in Cr (VI)–transformed cells (see Supplemental Material, Figure S3). Further investigation is needed to determine whether the same pathway is generally activated in other metal-induced cell transformation.

## Conculsions

The present study provides a new mechanism of arsenic-induced tumor angiogenesis and tumor growth. Arsenic-induced ROS inhibit miR-199a expression via DNMT1-mediated DNA methylation. The repression of miR-199a expression results in induction of HIF-1α and COX-2. In addition, the bidirectional regulations between HIF-1α and COX-2 forming a positive feedback further promote tumor angiogenesis and tumor growth (Figure 6C). Our findings may have clinical implication in targeted therapy for arsenic-induced lung cancer in the future.

## Supplemental Material

(483 KB) PDF
