# Supplementary material for: Chronic Arsenic Exposure and Angiogenesis in Human Bronchial Epithelial Cells via the ROS/miR-199a-5p/HIF-1α/COX-2 Pathway
Source: Environ Health Perspect. 2014 Jan 10;122(3):255–61. doi: 10.1289/ehp.1307545 (PMC3948041; doi:10.1289/ehp.1307545)
Supplement: (483 KB) PDF [file ehp.1307545.s001.pdf]

Environmental Health Perspectives doi:10.1289/ehp.1307545

Supplemental Material

Chronic Arsenic Exposure and Angiogenesis in Human Bronchial Epithelial Cells via the ROS/miR-199a-5p/HIF-1α/COX-2 Pathway

Jun He, Min Wang, Yue Jiang, Qiudan Chen, Shaohua Xu, Qing Xu, Bing-Hua Jiang, and Ling-Zhi Liu[2]

Table of Contents

Methods	page 2
Supplemental Material, Figure S1	page 3
Supplemental Material, Figure S2	page 4
Supplemental Material, Figure S3	page 6

Methods

RK3E cells transformation

RK3E cells were cultured in DMEM supplemented with 10% fetal bovine serum. N-Ras plasmid was kindly provided by J. M. Ruppert (West Virginia University, Morgantown, WV). The cDNA of N-Ras was then subcloned into the vector pBabe puro. Virus supernatant was obtained by serial transfecting package cells BOSC23 ecotropic cells (ATCC) and Am12 amphotropic cells (ATCC). RK3E cells were infected with N-Ras virus supernatant or pBabe-puro supernatant at a ratio of 2:1. The cells were used for transformation which was marked by focus formation in the culture for up to 10 days.

Supplemental figures and legends

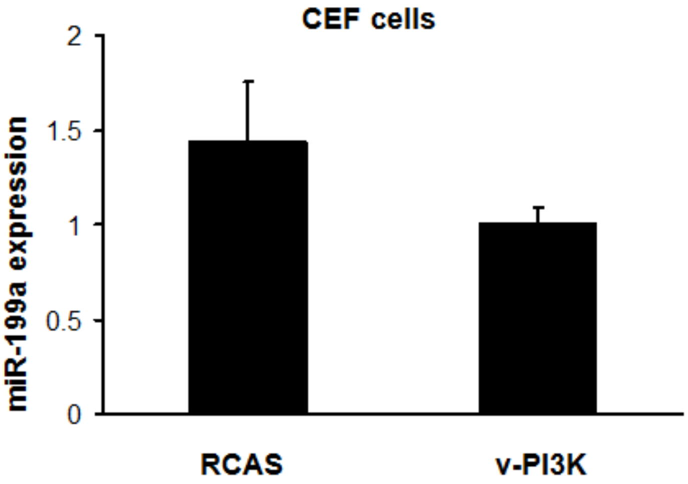

Figure S1. miR-199a expression in chicken embryo fibroblast cells (CEF) and PI3K-transformed CEF cells. The expression levels of miR-199a in parental CEF cells infected with retro-vector Replication Competent ALV LTR with a spiced acceptor (RCAS), and v-PI3K-transformed CEF cells were determined by RT-qPCR analysis. Relative miRNA expression levels were represented as Relative Quantification (RQ) using of $2^{-\Delta\Delta C_T}$ methods. The values were normalized to the U6 expression level. Mean ± SE values were from three separate experiments. There was no significant difference of miR-199a levels between two groups.

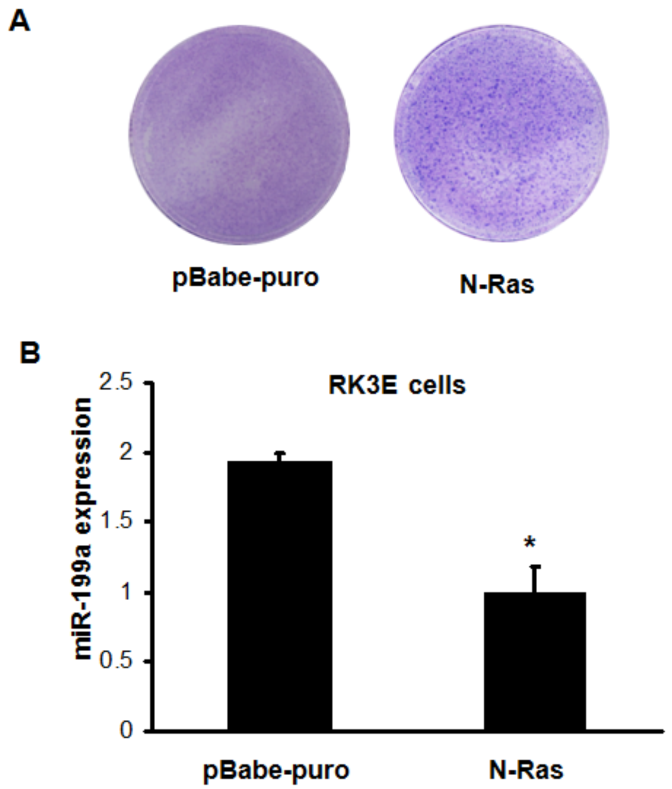

Figure S2. miR-199a expression levels in RK3E parental cells and N-Ras-transformed RK3E cells. (A) RK3E cells were infected with supernatant containing N-Ras virus or pBabe puro retrovirus. The cells were fixed and stained with Wright Stain (Sigma) 10 days in culture after the infection. Formation of foci, a marker of transformation, in RK3E cells following infection with N-Ras virus is shown. No focus was observed in vector control cells. (B) The expression levels of miR-199a in N-Ras-transformed cells and vector control cells were determined by Taqman RT-qPCR. Relative miRNA expression levels were represented as RQ using of $2^{-\Delta\Delta C_T}$ methods. The values were normalized to the U6 expression level. Mean ± SE values were obtained from three separate experiments. Student *t* test was used to compare the

difference between two groups. *indicates significant difference compared with vector control cells ($P < 0.05$).

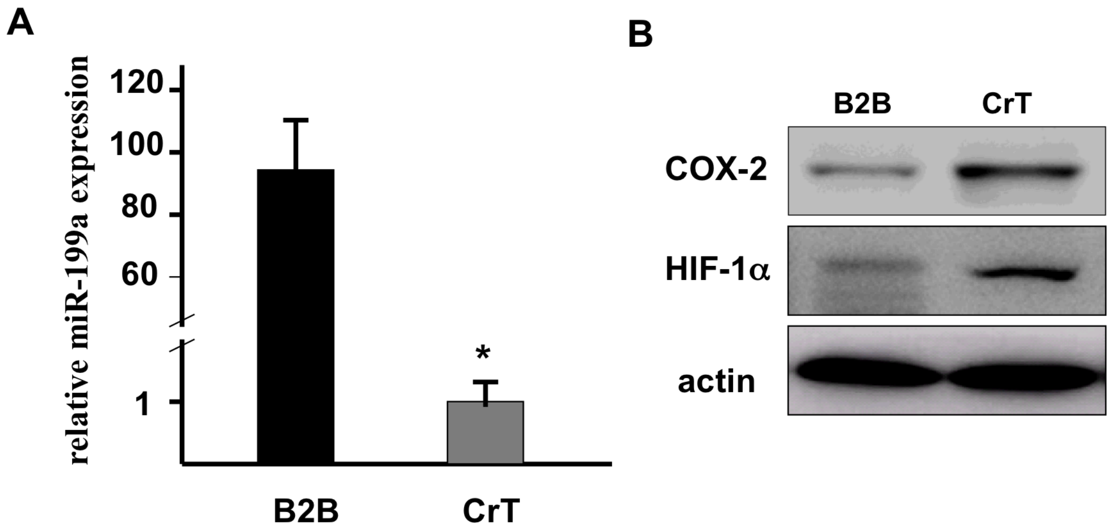

Figure S3. miR-199a, HIF-1α and COX-2 basal expression levels in chromium (VI)-transformed BEAS-2B cells. (A) To generate CrT cells, B2B cells were continuously cultured in DMEM medium containing 1 μM Cr (VI) for 6 months. Parallel cultures cultured in Cr (VI)-free medium acted as passage-matched controls. miR-199a expression levels in parental BEAS-2B cells (B2B) and chromium-transformed B2B (CrT) cells. Relative miRNA expression levels were represented as RQ using of $2^{-\Delta\Delta C_T}$ methods. The values were normalized to the U6 expression level. Mean ± SE values were obtained from three separate experiments. Student *t* test was used to compare the difference between two groups. * indicates significant difference compared with vector control cells ($P < 0.05$). (B) The basal protein levels of HIF-1α and COX-2 in B2B cells and CrT cells were determined by Western-blotting.
